# Emerging Infections and Pregnancy

**DOI:** 10.3201/eid1211.060152

**Published:** 2006-11

**Authors:** Denise J. Jamieson, Regan N. Theiler, Sonja A. Rasmussen

**Affiliations:** * Centers for Disease Control and Prevention, Atlanta, Georgia, USA;; † Emory University at Grady Health Systems, Atlanta, Georgia, USA

**Keywords:** emerging infectious diseases, pregnancy, immunology, synopsis

## Abstract

A key component of the response to emerging infections is consideration of special populations, including pregnant women. Successful pregnancy depends on adaptation of the woman's immune system to tolerate a genetically foreign fetus. Although the immune system changes are not well understood, a shift from cell-mediated immunity toward humoral immunity is believed to occur. These immunologic changes may alter susceptibility to and severity of infectious diseases in pregnant women. For example, pregnancy may increase susceptibility to toxoplasmosis and listeriosis and may increase severity of illness and increase mortality rates from influenza and varicella. Compared with information about more conventional disease threats, information about emerging infectious diseases is quite limited. Pregnant women's altered response to infectious diseases should be considered when planning a response to emerging infectious disease threats.

## Abstract 2

Immunologic changes of pregnancy may increase susceptibility to certain intracellular pathogens.

As strategies to deal with emerging infectious disease threats are developed, a key component is consideration of special populations, including pregnant women (*1*). Several issues are relevant to infectious disease threats during pregnancy. First, changes in immunity and physiology during pregnancy may make pregnant women more susceptible to or more severely affected by infectious diseases. Second, the effects of infectious diseases on the fetus may be unknown and difficult to predict, and diagnosis of infection in the fetus or infant can be challenging. Third, prophylaxis and treatment appropriate for the general population might not be appropriate for pregnant women. We focus on the first of these considerations: the immunology of pregnancy and the effects of emerging infectious diseases on the pregnant woman.

Although knowledge of the immunology of pregnancy has evolved tremendously over the past decade, many unanswered questions remain, such as how immune function is altered during pregnancy and how this alteration may affect susceptibility to and severity of infectious diseases. Although the effects of some infectious agents during pregnancy are well known, knowledge about many others is limited. A challenge to the study of infectious diseases during pregnancy is the selection of an appropriate control group; many studies have been retrospective and without control groups. Compared with knowledge about more conventional infectious disease threats, knowledge about novel and emerging infectious diseases during pregnancy is even more limited. Such lack of knowledge causes concern, given that an altered response to infectious diseases during pregnancy may require altered responses to emerging infectious disease threats. We describe the immunologic changes that may affect the course of infectious diseases in pregnant women, briefly summarize what is known about infectious diseases during pregnancy, and then focus on the particular challenges of dealing with emerging infectious diseases in pregnant women.

## Immunology of Pregnancy

One of the most intriguing puzzles in modern immunology involves the "paradox of pregnancy," in which immunologic tolerance to paternally derived fetal antigens is achieved despite an apparently adequate maternal defense against infection. With 50% of its genetic material derived from its father, the fetus's susceptibility to rejection by the maternal immune system is similar to the susceptibility of a transplanted organ. Evidence indicates that the maternal immune system may tolerate fetal antigens by suppressing cell-mediated immunity while retaining normal humoral immunity. These changes are known to occur locally at the maternal-fetal interface but may also affect systemic immune responses to infection. Although pregnant women are not immunosuppressed in the classic sense, immunologic changes of pregnancy may induce a state of increased susceptibility to certain intracellular pathogens, including viruses, intracellular bacteria, and parasites.

## Maternal-Fetal Interface

The fetal allograft is exposed to the maternal immune system at the placenta and fetal membranes (the amnion and chorion), collectively described as the maternal-fetal interface. On the fetal side of the interface, the placenta and membranes enclose the fetus and are derived entirely from fetal tissue. Forming a specialized epithelial surface within the placenta, fetal syncytiotrophoblast cells directly contact maternal blood for nutrient exchange. On the maternal side of the interface, the uterine tissue in contact with the placenta and fetal membranes, the decidua, is rich in specialized maternal immune cells including lymphocytes and macrophages (*2*). Despite the prolonged direct exposure of decidual leukocytes and maternal blood to fetal antigens, the immune system does not recognize the fetus as foreign. Several mechanisms underlie this maternal tolerance of fetal tissues.

## Humoral Immunity

Also known as antibody-mediated immunity, humoral immunity results from recognition of pathogens by specific antibodies. Most effective against extracellular pathogens, humoral immunity is essential for fighting many bacterial infections. The bacteria become coated in antibodies, which then mediate uptake of the pathogens by phagocytic cells, including neutrophils and macrophages. Presentation of the bacterial antigens on the surface of the macrophage then stimulates B lymphocytes specific to the pathogen, and the B cells produce more antibodies to control the infection. This humoral immune response is augmented by T-helper type II (Th2) lymphocytes, which provide costimulation and induce replication of the B cells. The Th2 response during pregnancy results in vigorous antibody-mediated immunity to pathogens (*2*).

## Cell-Mediated Immunity

Essential for controlling intracellular pathogens, cell-mediated immunity involves lymphocyte recognition of cell-associated foreign antigens, followed by destruction of the infected host cells. In contrast to humoral immunity, this arm of the immune response is stimulated by T-helper type I (Th1) lymphocytes and the cytokines they release. The most important effectors of the cell-mediated immune response, cytotoxic T lymphocytes, are the main immune cells that recognize foreign antigens on the surface of infected "self" cells. Cells infected with viruses or other intracellular pathogens are cytotoxic T lymphocytes' most common targets. The cell-mediated immune response is critical for controlling such pathogens because their intracellular location shelters them from antibody binding.

## T-Helper Cells and the Th1-Th2 Shift

Emphasis on cell-mediated immunity versus humoral immunity changes according to the type of T-helper lymphocytes responding to an infectious threat. Multiple factors, including the cytokine environment and costimulatory molecules present during activation of the T-helper cell, determine the development of either Th1- or Th2-helper phenotype. One hypothesis is that, in addition to hormonal factors that affect the Th1-Th2 balance, macrophages present at the maternal-fetal interface release predominantly Th2-stimulating cytokines and contribute to the overall dominance of humoral immunity during pregnancy (*3*). In addition to stimulating B lymphocytes, Th2 cells suppress the cytotoxic T lymphocyte response, decreasing the robustness of cell-mediated immunity. In the uterine decidua, the Th2 cytokine environment favors activation of B lymphocytes, resulting in stimulation of antibody secretion and suppression of cell-mediated immunity (*3*). This phenomenon is often referred to as the Th1-Th2 shift of pregnancy and is thought to contribute to maternal tolerance of the fetus by suppressing the antifetal cell-mediated immune response.

## Systemic Immune Changes

An evolving model of pregnancy-associated immune changes suggests that the hormonal environment of pregnancy contributes to local suppression of cell-mediated immunity at the maternal-fetal interface while mediating a systemic change toward Th2 dominance. That the local Th1-Th2 shift may also influence the systemic maternal immune response during pregnancy is evidenced in pregnant patients with autoimmune disorders. Women with rheumatoid arthritis, a predominantly cell-mediated autoimmune disorder, tend to experience remissions during pregnancy (*4*). Similarly, patients with multiple sclerosis have fewer exacerbations while pregnant but worsening symptoms during the postpartum period (*5*). Systemic lupus erythematosis, however, a predominantly antibody-mediated autoimmune disorder, often worsens during pregnancy, perhaps due to increased immunoglobulin synthesis and decreased clearance of immune complexes resulting from robust Th2 activity (*3**,**6*). These well-studied changes in severity of autoimmune disorders during pregnancy illustrate systemic immune alterations that occur in conjunction with the Th1-Th2 shift. Systemic suppression of cell-mediated immunity may contribute to increased susceptibility to some intracellular pathogens—including viruses, bacteria, and parasites—during pregnancy.

## Pregnancy and Conventional Infectious Disease Threats

Pregnant women may be more susceptible and more severely affected by several infectious diseases such as malaria and measles. Pregnant women in malaria-endemic regions are at risk of becoming infected with Plasmodium falciparum, 1 of 4 parasites that cause malaria in humans (*7*). The increased incidence and severity of malaria may occur especially in primiparous women. Although parasite density is highest in nonimmune women during their first pregnancy, even a previously immune woman can become more susceptible to malaria infection during pregnancy (*7*). In a 14-year follow-up study of women of reproductive age (15–45 years) in 1 area of the Gambia, McGregor and Smith found a higher prevalence of parasitemia among pregnant women than among nonpregnant women (*8*). Prevalence of infection and parasite density are highest during the first half of pregnancy and decline gradually during the second half (*7*).

Evidence also indicates that measles (rubeola) is more common and severe in pregnant women. Accounts of measles outbreaks before an effective vaccine was available indicate that pregnant women may be more severely affected. For example, the investigation of an outbreak of measles in Greenland in 1951 showed that mortality rates were higher among pregnant women than nonpregnant women. Pregnant women were also more likely to experience heart failure (*9*). A relatively recent outbreak of >1,700 confirmed cases of measles in Houston during 1988–1989 also resulted in a high rate of serious complications among infected pregnant women, which suggests that the outbreak disproportionately affected pregnant women (*10*).

## Increased Disease Susceptibility

Pregnancy may be a risk factor for acquiring certain infectious diseases, such as toxoplasmosis, Hansen disease, and listeriosis. Toxoplasma gondii is a parasite that infects humans primarily through ingestion of infected raw or undercooked meat and, less frequently, by exposure to infected cat feces. This intracellular pathogen can be transmitted transplacentally to the fetus. A cross-sectional study of 2,242 women in Brazil showed that previous pregnancy was a risk factor for serologic evidence of prior infection with toxoplasmosis (*11*). In a follow-up prospective cohort study, the same investigators found that pregnant women who were seronegative for Toxoplasma were more than twice as likely as nonpregnant women to seroconvert; acute infection developed in 8.6% of pregnant women (*12*). These findings are consistent with animal data showing that pregnant mice have lower resistance to Toxoplasma than nonpregnant control mice (*13*).

Pregnant women may be more likely to show clinical signs of Hansen disease, or leprosy. The causative agent, Mycobacterium leprae, can multiply and cause symptomatic disease, particularly in hosts with decreased immunity. The decreased cell-mediated immunity associated with pregnancy may predispose pregnant women to this disease (*14*). A recent report describes a cohort of 40 patients with Hansen disease in Texas, 3 of whom were pregnant (*14*). In addition to evidence supporting the theory that pregnant women are more susceptible to Hansen disease, evidence exists that pregnant women may be more likely to experience relapse of disease. Among 25 women in an Ethiopian cohort who had been treated and had therapy discontinued when considered cured, almost half (n = 12) experienced a relapse of disease when they became pregnant (*15*).

Listeria monocytogenes, a foodborne pathogen, is responsible for ≈2,500 cases of serious illness in the United States each year. Listeria infections are more common during pregnancy; one quarter to one third of all cases of listeriosis occur in pregnant women (*16**,**17*). In 2000, an outbreak of listeriosis among Hispanic persons in North Carolina was reported as a result of ingestion of contaminated homemade Mexican-style cheese; 11 of the 13 cases were in pregnant women (*18*).

## Increased Disease Severity

For pregnant women, certain infectious diseases, such as influenza and varicella, may have a more severe clinical course, increased complication rate, and higher case-fatality rate. For example, influenza infections cause more severe illness and higher mortality rates for pregnant women. During the 1918–19 influenza pandemic, the mortality rate was 27% for pregnant women, higher in the last trimester, and it increased to 50% if pneumonia developed (*19*). Freeman and Barno reported that during the 1957–1958 pandemic, 50% of the deaths from influenza among reproductive-aged women in Minnesota occurred in pregnant women and that influenza was the leading cause of maternal death in Minnesota (*20*). Increased incidence and severity of illness has also been observed during interpandemic periods. In a review of the Tennessee Medicaid program from 1974 through 1993, pregnant women in their third trimester were 3–4 times as likely as postpartum women to be hospitalized for an acute cardiopulmonary condition during influenza season (*21*). In addition to immunologic changes, other physiologic changes in pregnancy such as increased heart rate, stroke volume, and oxygen consumption, and decreased lung capacity may contribute to this increased risk for illness during pregnancy. Due to the high risk for influenza-related complications, women who will be pregnant during the influenza season should be vaccinated (*22*).

Clinical evidence indicates that primary varicella infections during pregnancy tend to be more severe and that varicella pneumonia seems to be more common among pregnant women than among nonpregnant women. For example, in a case-series of 43 pregnant women reported by Paryani and Arvin, pneumonia developed in ≈10%; 2 of these women required ventilatory support and 1 died (*23*). By comparison, the rate of pneumonia as a complication of varicella infection among the general population is 0.3%–1.8% (*24*). Similarly, pregnant women with varicella pneumonia are more likely to die than nonpregnant women with varicella pneumonia. Haake reviewed 34 published cases of untreated varicella pneumonia in pregnant women and found that 12 (35%) died. By contrast, the mortality rate for nonpregnant women with varicella is ≈11% (*24*).

## Challenges

Emerging infectious diseases, defined as infectious diseases whose incidence in humans has increased during the past 2 decades or threatens to increase in the near future, are increasingly recognized by physicians as an important threat to pregnant women. Emerging infectious diseases include novel pathogens that have newly emerged, such as severe acute respiratory syndrome (SARS), as well as pathogens that could potentially be used as biologic weapons. Unfortunately, information about how pregnant women are affected by many of these novel and emerging infections is limited.

## Novel Pathogens

During the worldwide outbreak of SARS in 2003, several countries reported cases in pregnant women. Although these numbers were too small to enable definitive conclusions as to whether SARS was more severe among pregnant than nonpregnant women, some evidence indicates that it may be. The largest case series of pregnant women with SARS was from Hong Kong Special Administrative Region, People's Republic of China, where 12 pregnant women with SARS were admitted to 5 public hospitals; 3 of them died, giving a case-fatality rate of 25% (*25*). In a case-control study conducted in the same region, pregnant women with SARS had more severe disease than nonpregnant women and an increased risk for admission to the intensive care unit, development of renal failure, development of disseminated intravascular coagulopathy, and death (*26*). Of 8 cases of laboratory-confirmed SARS reported in the United States, 2 were in pregnant women; the small number of cases precludes definitive conclusions about the severity of disease (*27*).

## Potential Effects of Bioterrorism

The Working Group on Civilian Biodefense has identified a limited number of biologic agents that are of particular concern (*28*). Evidence exists that infection with some of these pathogens, including smallpox virus and some of the hemorrhagic fever viruses, may be more severe during pregnancy.

Clinical experience with smallpox (variola virus) before vaccination and disease eradication indicates that pregnant women are more susceptible to variola infection and have more severe disease (*29**,**30*). Pregnancy is associated with an increased smallpox case-fatality rate; in the large case-series study in India reported by Rao et al., unvaccinated pregnant women were 3 times more likely to die than were nonpregnant women and men admitted to the hospital during the same time period (*29*). Pregnant women are more likely than nonpregnant women to have hemorrhagic smallpox (purpura variolosa), a severe variety of the disease (*30*).

The viral hemorrhagic fevers, including Lassa fever and Ebola, may be more severe during pregnancy. The first reported case of Lassa fever, caused by infection with an arenavirus, was described in a pregnant patient. In this initial outbreak, 11 patients and staff members who were exposed to the index patient died (*31*). The case-fatality rate is higher for pregnant women, particularly in the third trimester, than for nonpregnant women (*31**,**32*). Women who have Lassa fever late in pregnancy have the highest circulating levels of viremia and therefore tend to be the sickest. Evidence indicating that the placenta may be a preferred site for viral replication may help explain why illness and death increase during the third trimester of pregnancy (*32*). One study found that after pregnancy ended, whether by abortion or normal delivery, women rapidly improved (*32*).

Ebola virus, a member of the Filoviridae group, is transmitted by direct contact with blood, secretions, or contaminated objects and is associated with high case-fatality rates (*28*). Investigations of outbreaks in Africa suggest that Ebola infection may be more severe during pregnancy and that mortality rates are higher. Pregnant women infected with Ebola more often have serious complications, such as hemorrhagic and neurologic sequelae, than do nonpregnant patients (*31*). Unlike risk for death from Lassa fever, which is highest during the third trimester of pregnancy, risk for death from Ebola is similar during all trimesters (*33*).

## Other Emerging Infections

Pneumocystis jiroveci (formerly P. carinii) has long been identified as a cause of pneumonia in immunocompromised persons. Pneumocytis pneumonia was first identified in malnourished children in European orphanages during World War II and was later associated with severe immunosuppression in HIV-infected persons (*34*). However, this agent is increasingly causing infection among immunocompetent persons. A mild or asymptomatic form of P. jiroveci infection occurs in immunocompetent hosts, and this infection may be more common in pregnant women than in nonpregnant women. In a small pilot study, nasal swabs from 33 healthy women in their third trimester of pregnancy were compared with those from 28 healthy nonpregnant women. P. jiroveci DNA was isolated from 5 of the pregnant women and none of the nonpregnant women (p = 0.04) (*35*), which indicates that the immune changes associated with pregnancy may favor asymptomatic nasal carriage of this organism. Evidence also indicates that Pneumocytis pneumonia may be more severe during pregnancy (*35*) and that Pneumocystis may be perinatally transmitted by HIV-infected women to their children (*34*).

Psittacosis is primarily a flulike illness characterized by fever, headache, and atypical pneumonia. Chlamydophila psittaci (formerly Chlamydia psittaci), the causative agent, is transmitted by inhalation of material from infected birds or by exposure to infected amniotic fluid or placentas of sheep or goats. Although each year, ≈75–100 cases of psittacosis occur in the United States, only 14 cases of psittacosis have been reported in pregnant women, including a recent case in a pregnant Montana sheep rancher. Illness during pregnancy can be quite severe, mimicking HELLP (hemolysis, elevated liver enzyme levels, and low platelet count) syndrome but without hypertension. Most women rapidly recover after pregnancy (*36*).

## Conclusions

Changes in immune function during pregnancy alter a pregnant woman's susceptibility to and severity of certain infectious diseases. These alterations are particularly problematic because physicians may hesitate to provide prophylaxis or aggressive treatment to pregnant women because of concerns about effects on the fetus. For example, despite the 1997 recommendation that women who would be in their second or third trimester of pregnancy during influenza season receive the inactivated influenza vaccine, among women 18–44 years of age, reports of having received the influenza vaccination during the past 12 months were fewer for pregnant than for nonpregnant women (*37*).

Compared with what is known about conventional disease threats, knowledge about currently recognized emerging infectious diseases is quite limited. Soon we will likely be faced with novel pathogens about which little or nothing is known. Because the effects of emerging infections in pregnant women might differ from those in the general population, pregnancy must be considered a potential risk factor for disease susceptibility as well as for illness and death. Unfortunately, pregnancy issues are often not well addressed in outbreak investigations, ongoing prospective studies, or emergency preparedness planning. Future scientific inquiry and medical investigations must include pregnancy-related issues as a vital component.
